# Rhythms in cortisol mediate sleep and circadian impacts on health

**DOI:** 10.1093/sleep/zsae151

**Published:** 2024-07-04

**Authors:** Peter Y Liu

**Affiliations:** Division of Endocrinology, Department of Medicine, David Geffen School of Medicine at UCLA, Harbor-UCLA Medical Center and Genomics Institute, The Lundquist Institute, Torrance, CA, USA

Multicellular organisms developed timing systems (i.e. cellular clocks) to synchronize external nutrient cycles that follow an environmental 24-hour rhythm with internal cycles: energetic processes controlling metabolism, restorative activities including sleep, and reproductive programs which have a metabolic cost, but are necessary for survival. In mammals, cellular clocks in peripheral organs are coordinated through hormonal and neural signals orchestrated by the central circadian pacemaker (CCP) located in the suprachiasmatic nucleus of the hypothalamus [1]. These endogenous hormonal signals exhibit 24-hour rhythms in the blood that are recognizable in the absence of external cycles of light–dark, wake–sleep, feeding–fasting, and activity–rest. In humans, the constant routine protocol experimentally removes or uniformly distributes such influences to separate endogenous hormone rhythms from those due to exogenous cycles [2, 3]. Three such experiments utilizing constant routine have confirmed that the 24-hour rhythm in cortisol is driven by the CCP and that the cortisol rhythm peaks at the habitual sleep–wake transition in the morning, and progressively decreases to a nadir in the evening [4–6]. This rhythm is generally believed to be the central *metabolic* synchronizing signal of the CCP for metabolically important tissues such as liver, muscle, and adipose tissue which are the major storage sites for glycogen, protein, and fat, respectively [7–9]. The synchronizing effect of cortisol on peripheral clocks in these tissues is direct and mediated by glucocorticoid response elements present in regulatory regions of core clock genes because effects are generally lost when such response elements are knocked out by molecular manipulations. Interestingly, cortisol also appears to synchronize cellular clocks in other systems that are metabolically intensive, but necessary for survival such as the Leydig cell which synthesizes testicular testosterone and thereby maintains spermatogenesis (Figure 1).

**Figure 1.**
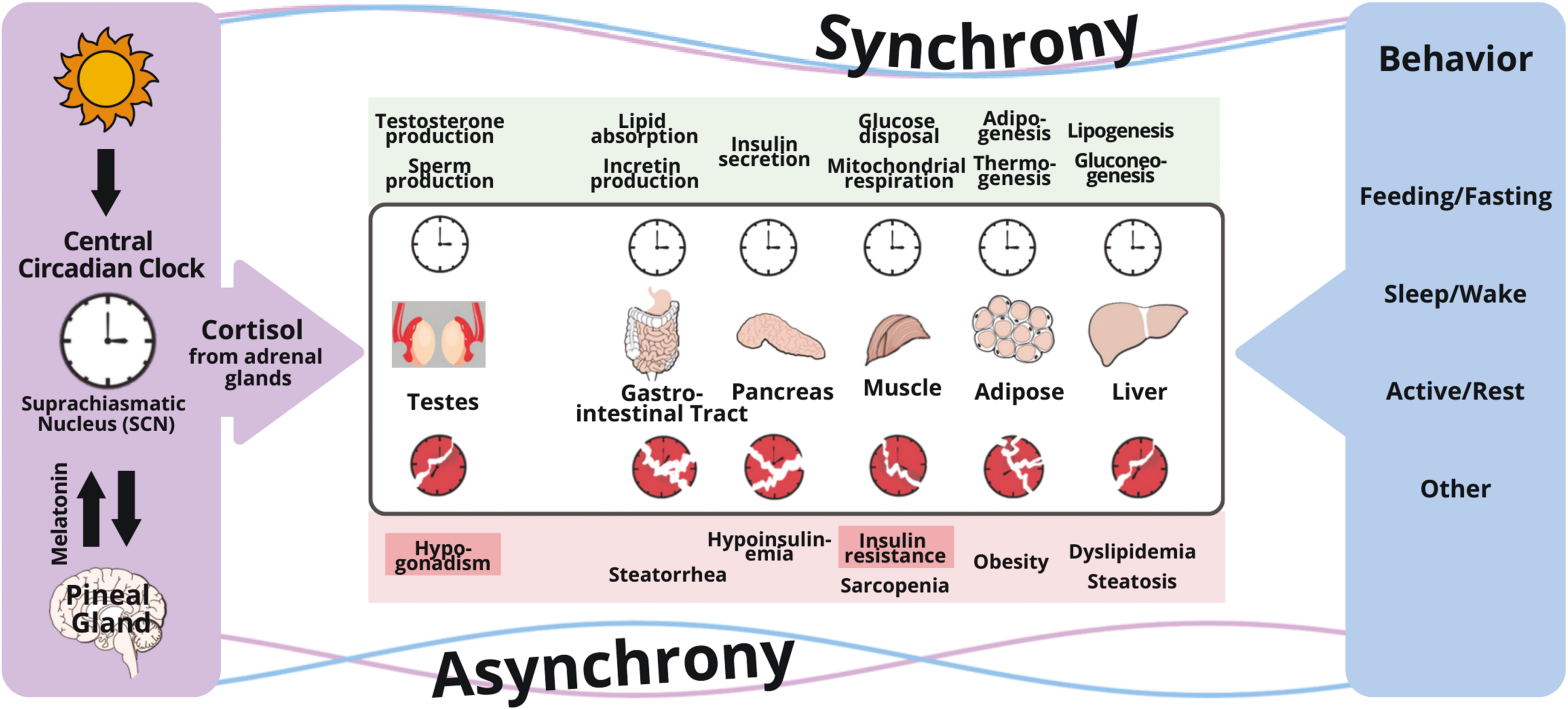
Conceptual framework for the disruption of the circadian regulation of clocks in peripheral organs by conflicting signaling from the central circadian clock and behavioral cycles (feeding–fasting, sleep–wake, and activity–rest). The central timing signal is cortisol for Leydig cells in the testes, and peripheral organs responsible for whole-body metabolism (gut, pancreas, muscle, fat, and liver), whereas peripheral signals governed by behavior include glucagon, insulin, other hormones, metabolites, and others. When peripheral clocks receive conflicting signals, clocks are disrupted and desynchrony occurs, which leads to insulin resistance and other pathologies.

In addition to timing, a flatter diurnal slope, which represents a slower decline in cortisol to the nadir, is widely believed to mediate many mental and physical disease processes including from insufficient sleep or aging [7, 10]. Interventional studies conducted in controlled laboratory environments support these assertions and show an increase in late afternoon–early evening cortisol (which is analogous to flattening of the diurnal cortisol slope), but no change in 24-hour mean cortisol with sleep loss [7]. These cortisol changes are in conjunction with a decrease in 24-hour testosterone in men [11]. Preventing both the flattening of the diurnal slope of cortisol and the reduction in testosterone in men by means of a dual hormone clamp mitigates the development of insulin resistance—which triggers diabetes mellitus—from sleep restriction by at least 50% [12]. This interventional study thereby directly identifies abnormal cortisol (i.e. catabolic) and testosterone (i.e. anabolic) signaling as major mechanisms by which insufficient sleep leads to metabolic harm. Although future studies are needed to separate cortisol from testosterone effects, these findings provide proof that targeting catabolic–anabolic signaling pathways may be a pathophysiologically informed approach to substantially mitigate metabolic harm from sleep loss without requiring more sleep. Developing such methods are needed because sleeping more is not possible for many individuals due to occupational and life demands, and mitigating insulin resistance will yield important societal benefits and healthcare savings for common and costly metabolic diseases such as diabetes mellitus [13, 14].

Although such studies conducted in the chronobiology laboratory generate important mechanistic insights, complementary in-the-field naturalistic studies are needed to assess potential bidirectional relationships in larger cohorts for longer periods of time. Yap and colleagues conducted such a naturalistic study [15]. Ninety-five young healthy predominately Asian women relocating to Australia for tertiary education provided two saliva samples (within 2 hours of habitual awakening and within 4 hours of habitual sleep time) each day for 14 consecutive days, in conjunction with simultaneous at-home EEG sleep recording for 15 consecutive nights and contemporaneously collected daily reports of events and activities by smartphone app. Cortisol was later measured in about 2000 viable time-stamped saliva samples and these cortisol concentrations were related to over 900 nights of sleep monitoring. Longitudinal analyses showed that higher presleep cortisol predicted shorter subsequent total sleep time, lower sleep efficiency, and longer sleep onset latency that night; and that shorter and poorer sleep was associated with flatter diurnal cortisol slope the next day [15]. These associations point to bidirectional relationships that need to be further investigated experimentally through actual or Mendelian randomization.

Although the effect sizes of these relationships were small, seemingly minor changes in cortisol patterns which are nominally within the cortisol reference range can have important metabolic effects in other circumstances. For example, cortisol delivery that replicates the proper diurnal slope provides additional metabolic benefits (optimized weight, blood pressure, and glucose metabolism) than delivery which results in a flatter diurnal slope, even when the total dose of cortisol delivered is identical under the two conditions [16, 17]. Yap et al. also demonstrated the feasibility and potential scalability of conducting large-scale, longitudinal naturalistic studies of sleep and cortisol to unveil bidirectional relationships. Such investigations are needed because sleep influences health and longevity through endocrine and metabolic systems [18]. The methods utilized could also be applied to study even longer-term infradian rhythms of cortisol. Seasonal changes in reproduction (timed to optimize reproduction which is metabolically costly to longer-term environmental energetic cycles governed by season) and the monthly menstrual cycle are examples of infradian rhythms that have been little studied in relation to cortisol.

Ultradian (i.e. pulsatile) rhythms in cortisol also exist, and these pulses allow for rapid responses that fine-tune the body’s reaction to unpredictable external and internal insults [7, 19, 20]. Salivary cortisol, as utilized by Yap et al. [15], summates and does not capture these pulses because they occur too frequently every 90 minutes. Other technological advances are therefore needed to fully appreciate the relationships among ultradian, diurnal, and infradian hormonal rhythms (including those of cortisol), sleep and health and longevity. Understanding pulsatility is important in this context because specific pulse characteristics of cortisol are crucial for signaling [21]. And are altered by experimental sleep deprivation in a time-of-day dependent manner [22]. These pulses are not regulated by the CCP because sectioning of the suprachiasmatic nucleus does not ablate pulsatility. In fact, ultradian cortisol rhythms are regulated by the hypothalamo-pituitary adrenal axis which governs stress responses, catabolism, immune function, and other homeostatic processes through pulsatile feedforward (stimulatory) and feedback (inhibitory) signaling among its hormonal and neural elements [7, 19]. The regulation of cortisol by this highly integrated network is specific to the steroidogenic cells of the fasciculata (middle zone) of the adrenal cortex, which develops from centripetal transdifferentiation under opposing gradients of canonical WNT signaling which promotes glomerulosa (outer zone) formation, and protein kinase A signaling which promotes reticularis (inner zone) formation [23].

Technological advances are occurring to make near-continuous cortisol monitoring a reality [24, 25]. Such advances could revolutionize the assessment and management of cortisol disorders, much like how coin-shaped continuous glucose monitors that are inconspicuously worn and highly accurate, are revolutionizing the management of diabetes mellitus. More naturalistic studies to properly document cortisol rhythms in health and disease are needed. Once identified, properly replicating the ultradian and circadian properties of these rhythms to optimize hormone replacement will represent a major advance in the field of endocrinology. Such methods, utilizing subcutaneous delivery of cortisol are already being developed [26].

## References

[1] Bass J , Takahashi JS. Circadian integration of metabolism and energetics. Science. 2010;330(6009):1349–1354. doi: 10.1126/science.1195027 21127246 PMC3756146

[2] Duffy JF , Dijk DJ. Getting through to circadian oscillators: why use constant routines? J Biol Rhythms. 2002;17(1):4–13. doi: 10.1177/074873002129002294 11837947

[3] Mills JN , Minors DS, Waterhouse JM. Adaptation to abrupt time shifts of the oscillator(s) controlling human circadian rhythms. J Physiol. 1978;285:455–470. doi: 10.1113/jphysiol.1978.sp012582 745108 PMC1281767

[4] Kelly MR , Yuen F, Satterfield BC, et al. Endogenous diurnal patterns of adrenal and gonadal hormones during a 24-hour constant routine after simulated shift work. J Endocr Soc. 2022;6(12):bvac153. doi: 10.1210/jendso/bvac153 36330292 PMC9620969

[5] Uchiyama M , Ishibashi K, Enomoto T, et al. Twenty-four hour profiles of four hormones under constant routine. Psychiatry Clin Neurosci. 1998;52(2):241–243. doi: 10.1111/j.1440-1819.1998.tb01053.x 9628174

[6] Wright KP, Jr, Drake AL, Frey DJ, et al. Influence of sleep deprivation and circadian misalignment on cortisol, inflammatory markers, and cytokine balance. Brain Behav Immun. 2015;47:24–34. doi: 10.1016/j.bbi.2015.01.004 25640603 PMC5401766

[7] Liu PY , Reddy RT. Sleep, testosterone and cortisol balance, and ageing men. Rev Endocr Metab Disord. 2022;23:1323–1339. doi: 10.1007/s11154-022-09755-4 36152143 PMC9510302

[8] Oster H , Challet E, Ott V, et al. The functional and clinical significance of the 24-hour rhythm of circulating glucocorticoids. Endocr Rev. 2017;38(1):3–45. doi: 10.1210/er.2015-1080 27749086 PMC5563520

[9] Wu T , Yang L, Jiang J, et al. Chronic glucocorticoid treatment induced circadian clock disorder leads to lipid metabolism and gut microbiota alterations in rats. Life Sci. 2018;192:173–182. doi: 10.1016/j.lfs.2017.11.049 29196049

[10] Adam EK , Quinn ME, Tavernier R, McQuillan MT, Dahlke KA, Gilbert KE. Diurnal cortisol slopes and mental and physical health outcomes: a systematic review and meta-analysis. Psychoneuroendocrinology. 2017;83:25–41. doi: 10.1016/j.psyneuen.2017.05.018 28578301 PMC5568897

[11] O’Byrne NA , Yuen F, Niaz W, Liu PY. Sleep and the testis. Curr Opin Endocr Metab Res. 2021;18:83–93. doi: 10.1016/j.coemr.2021.03.002 33937581 PMC8087280

[12] Liu PY , Lawrence-Sidebottom D, Piotrowska K, et al. Clamping cortisol and testosterone mitigates the development of insulin resistance during sleep restriction in men. J Clin Endocrinol Metab. 2021;106(9):e3436–e3448. doi: 10.1210/clinem/dgab375 34043794 PMC8660069

[13] Parker ED , Lin J, Mahoney T, et al. Economic costs of diabetes in the U.S. in 2022. Diabetes Care. 2024;47(1):26–43. doi: 10.2337/dci23-0085 37909353

[14] ElSayed NA , Aleppo G, Aroda VR, et al; on behalf of the American Diabetes Association. 3. prevention or delay of type 2 diabetes and associated comorbidities: standards of care in diabetes-2023. Diabetes Care. 2023;46(suppl 1):S41–S48. doi:10.2337/dc23-S003 36507633 PMC9810464

[15] Yap Y , Tung NYC, Shen L, Bei B, Phillips A, Wiley JF. Daily associations between salivary cortisol and EEG-Assessed Sleep: A 15-Day Intensive Longitudinal Study. Sleep. 2024;47(9):1–10. doi: 10.1093/sleep/zsae087 PMC11381568 38587464

[16] Johannsson G , Nilsson AG, Bergthorsdottir R, et al. Improved cortisol exposure-time profile and outcome in patients with adrenal insufficiency: a prospective randomized trial of a novel hydrocortisone dual-release formulation. J Clin Endocrinol Metab. 2012;97(2):473–481. doi: 10.1210/jc.2011-1926 22112807

[17] O’Byrne NA , Yuen F, Butt W, Liu PY. Sleep and circadian regulation of cortisol: a short review. Curr Opin Endocr Metab Res. 2021;18:178–186. doi: 10.1016/j.coemr.2021.03.011 35128146 PMC8813037

[18] Carroll JE , Liu PY. Editorial overview: sleep as essential for health and longevity via endocrine and metabolic regulated systems. Curr Opin Endocr Metabol Res. 2021;18:v–vii. doi: 10.1016/j.coemr.2021.04.004

[19] Russell G , Lightman S. The human stress response. Nat Rev Endocrinol. 2019;15(9):525–534. doi: 10.1038/s41574-019-0228-0 31249398

[20] Veldhuis JD , Keenan DM, Pincus SM. Motivations and methods for analyzing pulsatile hormone secretion. Endocr Rev. 2008;29(7):823–864. doi: 10.1210/er.2008-0005 18940916 PMC2647703

[21] Lightman SL , Birnie MT, Conway-Campbell BL. Dynamics of ACTH and cortisol secretion and implications for disease. Endocr Rev. 2020;41(3):470–490. 10.1210/endrev/bnaa002 PMC7240781 32060528

[22] Liu PY , Takahashi PY, Yang RJ, Iranmanesh A, Veldhuis JD. Age and time-of-day differences in the hypothalamo-pituitary-testicular, and adrenal, response to total overnight sleep deprivation. Sleep. 2020;43(7). doi: 10.1093/sleep/zsaa008 PMC7355405 31993665

[23] Lyraki R , Schedl A. Adrenal cortex renewal in health and disease. Nat Rev Endocrinol. 2021;17(7):421–434. doi: 10.1038/s41574-021-00491-4 34011989

[24] Upton TJ , Zavala E, Methlie P, et al. High-resolution daily profiles of tissue adrenal steroids by portable automated collection. Sci Transl Med. 2023;15(701):eadg8464. doi: 10.1126/scitranslmed.adg8464 37343084

[25] Vignesh V , Castro-Dominguez B, James TD, Gamble-Turner JM, Lightman S, Reis NM. Advancements in cortisol detection: from conventional methods to next-generation technologies for enhanced hormone monitoring. ACS Sens. 2024;9(4):1666–1681. doi: 10.1021/acssensors.3c01912 38551608 PMC11059103

[26] Russell G , Kalafatakis K, Durant C, et al. Ultradian hydrocortisone replacement alters neuronal processing, emotional ambiguity, affect and fatigue in adrenal insufficiency: The PULSES trial. J Intern Med. 2024;295(1):51–67. doi: 10.1111/joim.13721 37857352 PMC10952319

